# Development of Rapid and Visual Nucleic Acid Detection Methods towards Four Serotypes of Human Adenovirus Species B Based on RPA-LF Test

**DOI:** 10.1155/2021/9957747

**Published:** 2021-10-04

**Authors:** Yong Qi, Wei Li, Xiaoling Li, Wanpeng Shen, Jinhai Zhang, Jiameng Li, Ruichen Lv, Nianhong Lu, Liqiang Zong, Susu Zhuang, Qiyuan Gui, Dongming Zhou, Jing Li, Yingjia Xu, Hongbing Shen, Yuexi Li

**Affiliations:** ^1^Huadong Research Institute for Medicine and Biotechniques, 210002, #293 Zhongshandonglu, Nanjing, Jiangsu, China; ^2^Nanjing Medical University, 211166, 101 Longmian Avenue, Jiangning District, Nanjing, Jiangsu, China; ^3^China Pharmaceutical University, 210009, #24 Tongjiaxiang, Nanjing, Jiangsu, China

## Abstract

**Objectives:**

Human adenoviruses (HAdV) are classified as 7 HAdV species, and some serotypes in species B like HAdV 3, HAdV 7, HAdV 21, and HAdV 55 caused severe symptoms, even fatalities. Patients may be misdiagnosed and inadequately treated without reliable and practical methods for HAdV serotyping. Developing rapid, sensitive, and specific diagnostic methods for HAdV is critical.

**Methods:**

Detection methods were established based on a recombinase polymerase amplification (RPA) assay and lateral flow (LF) test. Specific target sequence was screened, targeting which, primers and probes were designed, synthesized, and screened for establishing assay with high amplification efficiency. Primer or probe concentrations and amplification time were optimized. Detection limit, sensitivity, and specificity were evaluated. *Results and Conclusions*. Simple, sensitive, and specific RPA-LF methods for detection of four serotypes of HAdV together or separately were established, which had detection limits of 10 to 280 copies/reaction comparable to real-time PCR without recognizing other pathogens. The sensitivity and specificity were >92% and >98%, respectively, evaluated by limited clinical samples. The detection can be completed in 25 min without requirement of any instrument except a constant temperature equipment, showing superior detection performance and promising for a wide use in the field and resource-limited area.

## 1. Introduction

Human adenoviruses (HAdV) are nonenveloped double-stranded DNA viruses, which are classified as 7 HAdV species (HAdV-A to HAdV-G) including no less than 100 serotypes [1, 2]. Different HAdV serotypes have been associated with different clinical syndromes such as fever, acute respiratory disease (ARD), gastroenteritis, and conjunctivitis [3]. Among these serotypes, HAdV 3 and HAdV 7 are the main causative pathogens of lower respiratory tract infection epidemics that cause fatalities [4, 5] and are epidemic in many countries [6], including Singapore, the United States, the United Kingdom, Korea [7], and Canada [8]. In China, except for HAdV 3 and HAdV 7 [9, 10], HAdV 21 [11] and HAdV 55 [12, 13] that also cause severe symptoms and belong to species B were reported sporadically.

HAdV are one of the major pathogens associated with approximately 5% to 10% of the lower respiratory tract infections in infants and children [14, 15]. Patients may be misdiagnosed and inadequately treated without reliable and practical methods for HAdV typing [16]. Therefore, it is critical to develop rapid, sensitive, and specific diagnostic method for HAdV. Traditional detection methods, such as viral culture, are time-consuming and laborious [17]. Serologic tests rely on the antibody generating time and are frequently negative at the beginning of the disease. Commonly used molecular diagnostic assays, such as polymerase chain reaction (PCR) [18], real-time PCR [19, 20], and nested PCR [21], perform high sensitivity and specificity. However, they require a thermal cycler and well-trained specialists. These methods are not suitable for rapid disease diagnosis in the field or resource-limited areas. Compared with traditional methods, isothermal nucleic acid amplification methods such as loop-mediated isothermal amplification (LAMP) [22] are simple, rapid, and cost-effective.

Recently, a new isothermal amplification method named recombinase polymerase amplification (RPA) assay, which relies on a recombinase, a polymerase, and a single strand binding protein, was developed and could complete amplification in 5-20 min at around 37°C with a detection limit of lower than 10 copies/reaction [23]. Moreover, when combining RPA with lateral flow (LF) test, the target sequence can be detected in a simple, fast, and visual way that is useful in the field and resource-limited areas. In the present study, such universal and typing detection methods towards four serotypes of HAdV were established and evaluated.

## 2. Materials and Methods

### 2.1. Ethics Statement and Sample Preparation

A total of 200 clinical samples (throat swabs) from patients (age from 17 to 50) with febrile respiratory syndrome were collected from 2015 to 2017. They were stored at −80°C until extraction of nucleic acids using a MiniBEST Viral RNA/DNA Extraction Kit (Takara, Beijing, China). These specimens had previously been tested using real-time PCR or real-time RT-PCR kit for respiratory disease, including Influenza Virus A Real-Time RT-PCR Reagent (Shanghai ZJ Bio-Tech Corp, Shanghai, China) and Respiratory Adenovirus Real-Time PCR Kit (Shanghai ZJ Bio-Tech Corp). Then, they were tested using corresponding real-time PCR/RT-PCR kit for various types from the same company. Fifteen specimens were found to be positive for HAdV 3, 89 specimens positive for HAdV 7, 17 specimens positive for HAdV 55, and 79 specimens positive for influenza virus A (41 H1 positive and 38 H3 positive).

HAdV 21 DNA-spiked human samples were prepared to simulate patient samples for lack of actual patient samples. Briefly, throat swab samples were collected from healthy volunteers. Various concentrations of genomic DNA of HAdV 21 were mixed with each of 200 *μ*L of the samples to make DNA-spiked samples. DNA of the spiked samples were extracted using a MiniBEST Viral RNA/DNA Extraction Kit (Takara) as described previously.

Genomic DNA of *Coxiella burnetii* and *Chlamydia psittaci* were kindly given by Professor Bohai Wen and Lihua Song from the State Key Laboratory of Pathogens and Biosafety of China. Genomic DNA of *Streptococcus suis*, *Escherichia coli*, and *Staphylococcus aureus* was extracted from the corresponding bacteria using a QIAamp Blood and Tissue Mini DNA kit (Qiagen, CA, USA). For quality control, the existence of the corresponding genomic DNA in the samples was detected using real-time PCR methods as described previously [24].

The use of human samples was approved by the Ethics Committee of Huadong Research Institute for Medicine and Biotechniques, and consent form was signed.

### 2.2. Primers and Probe Design and Synthesis

Hexon gene from each serotype (GenBank AB330084.1 for HAdV 3; GenBank AC_000018.1 for HAdV 7; GenBank AB053166.1 for HAdV 21; and GenBank KF911353.1 for HAdV 55) was selected as target sequence and aligned using Multiple Sequence Alignment function of the DNAman software. The conserved sequence (about 300 bp) with high identical rate among hexon genes from those serotypes was used as target to design primers and probes to establish universal RPA assay (RPA-HAdV), which could detect all the four serotypes. It is difficult to screen serotype-specific sequences with lengths of over 100 bp as targets, considering that different serotypes have very minute variations in terms of their genetic organization. So the serotype-specific sequences with lengths of 30 to 50 bp were screened for specific probe design, and the sequences about 200 bp upstream and downstream of the specific sequence were used as targets to establish typing RPA assay for HAdV 3 (RPA-HAdV3), HAdV 7 (RPA-HAdV7), HAdV 21 (RPA-HAdV21), and HAdV 55 (RPA-HAdV55), respectively.

The probes (46 bp) were designed using Primer Premier 5 software (PREMIER Biosoft International, CA, USA). Several sets of primers were designed upstream and downstream of the probe. The 5′ end of the probes was labeled with carboxyfluorescein (FAM), the 3′ end blocked with a phosphate group, and a base analog tetrahydrofuran (THF) inserted to replace the 31st base. The 5′ end of the reverse primers was labeled with biotin. All the primers and probes were synthesized by GenScript company (Nanjing, China).

### 2.3. Positive Plasmid Construction

The hexon gene sequence of each serotype was either amplified using corresponding primers (Table 1) and genomic DNAs (for HAdV 3, 7, or 55) or directly synthesized by GenScript company (for HAdV 21). A 25 *μ*L of PCR reaction system with 12.5 *μ*L of 2x PCR Mix (Takara), 1.5 *μ*L forward or reverse primer (10 nM), 7.5 *μ*L of ddH2O, and 2 *μ*L of template DNA was used to amplify the target DNA. The amplification product was analyzed by agarose gel electrophoresis, purified using a SanPrep Agarose Gel DNA Purification Kit (Sangon, Shanghai, China), and ligated to pMD 18-T using a pMD 18-T vector cloning kit (Takara). The recombinant plasmids HAdV3-Hexon-pMD18T, HAdV7-Hexon-pMD18T, and HAdV55-Hexon-pMD18T were transformed into DH5*α E. coli* cells and scrubbed onto solid LB culture with ampicillin, X-Gal, and IPTG added. The white bacterial colony was picked up, and the existence of the target recombinant plasmids was confirmed using PCR as described above. The synthesized hexon gene of HAdV 21 was ligated to pUC 57 plasmid to construct a recombinant plasmid HAdV21-Hexon-pUC57, which was then transformed into DH5*α E. coli* cells as described previously [24]. The recombinant plasmid HAdV21-Hexon-pUC57 was digested by both *Eco*R I and *Hind* III and analyzed by gel electrophoreses to verify its successful construction. All the recombinant plasmids were purified, sequenced, and stored at −80°C before use.

### 2.4. Establishment of RPA-LF Assay

For RPA assay, each reverse primer and probe (Table 1) were combined to make various groups and the best one was screened using a preliminary RPA reaction system recommended in the commercial TwistAmp® RPA nfo kit (TwistDx Limited, Cambridge, UK). Briefly, 2.1 *μ*L of forward primer (10 *μ*M), 2.1 *μ*L of reverse primer (10 *μ*M), 0.6 *μ*L of probe (10 *μ*M), 1 *μ*L of template (1.66 × 10^1^ fM or 1 × 10^4^ copies/*μ*L), 12.2 *μ*L of DNase- and RNase-free water, and 29.5 *μ*L of rehydration buffer (provided in the kit) were mixed together to rehydrate the enzyme pellet (provided in the kit). Then, 2.5 *μ*L of MgAc (280 mM) was added to initiate the reaction following with incubation at 37°C for 20 min. For the universal RPA assay RPA-HAdV, the recombinant plasmids HAdV3-Hexon-pMD18T and HAdV55-Hexon-pMD18T were used as templates, and for the typing RPA assays, the corresponding recombinant plasmids were used.

After the reaction, the entire reaction tube was inserted into a cross-contamination-proof (XCP) lateral flow cassette (BioUstar, Hangzhou, China) and the cassette was closed as per the manufacturer's instruction. Results were judged visually by the naked eyes. A positive result was determined when both test line (T line) and control line (C line) developed, indicating sequences labeled with both FAM and biotin existed in the product. Only C line developing indicated a negative result, and if C line did not develop, the cassette should be replaced.

### 2.5. Reaction Condition Optimization

The concentrations of reverse primer and probe and the amplification time in each RPA assay were optimized. Briefly, various concentrations (10 *μ*M, 5 *μ*M, or 2.5 *μ*M) of reverse primer and probe were used to conduct the RPA-LF method as mentioned above, and the best concentration combination was determined through the developing strips in the cassette. Moreover, various amplification times (10 min, 15 min, or 20 min) were also evaluated. Positive or control plasmid at a concentration of 1 × 10^4^ copies/*μ*L was used as template in the reaction.

### 2.6. Detection Limit Evaluation

The positive plasmids were diluted into serial dilutions from 1 × 10^4^ to 1 × 10^0^ copies/*μ*L and used as templates to evaluate the detection limit in detection of positive plasmids.

Genomic DNA of various serotypes of HAdV was diluted into serial dilutions, and their concentrations were determined using quantitative PCR (qPCR) as described previously [25]. The primers and probe used in qPCR are indicated in Table 1. Then, the serial dilutions were used as templates to evaluate the detection limit of the optimized RPA-LF methods in detection of genomic DNA.

The detection limit was determined as the concentration of the highest dilution that gave a positive detection result. The evaluation with each dilution of positive plasmid or genomic DNA was done in duplicate.

### 2.7. Specificity and Sensitivity Evaluation

To determine the specificity of the established RPA-LF assays, their cross-reaction possibilities with genomic DNA of other pathogens, including various serotypes of HAdV, *C. psittaci*, *C. burnetii*, *S. suis*, *E. coli*, and *S. aureus*, were tested. Also, the extracted nucleic acids from 200 clinical samples and 10 HAdV 21 DNA-spiked samples were used as templates to determine the specificity and sensitivity of the established methods.

## 3. Results

### 3.1. Construction of Positive Plasmids

**Figure 1:**
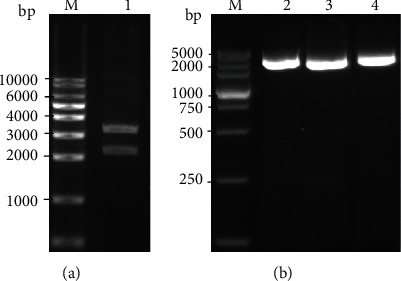
Verification of the positive plasmids with (a) double-enzyme digestion or (b) PCR following gel electrophoreses. Lane M: DNA marker; lane 1: plasmid HAdV21-Hexon-pUC57 digested by *Eco*R I and *Hind* III; lanes 2 to 4: PCR results with plasmids HAdV3-Hexon-pMD18T, HAdV7-Hexon-pMD18T, and HAdV55-Hexon-pMD18T as templates. The size is indicated on the left.

Positive plasmid for each serotype was constructed and verified. HAdV21-Hexon-pUC57 was digested by enzymes to verify its successful construction. As shown on Figure 1(a), the target sequence was exactly the same size as expected. The successful constructions of HAdV3-Hexon-pMD18T, HAdV7-Hexon-pMD18T, and HAdV55-Hexon-pMD18T were verified by PCR, and the amplified products were also exactly the same size as expected (Figure 1(b)).

### 3.2. Design of Primers and Probes

**Figure 2:**
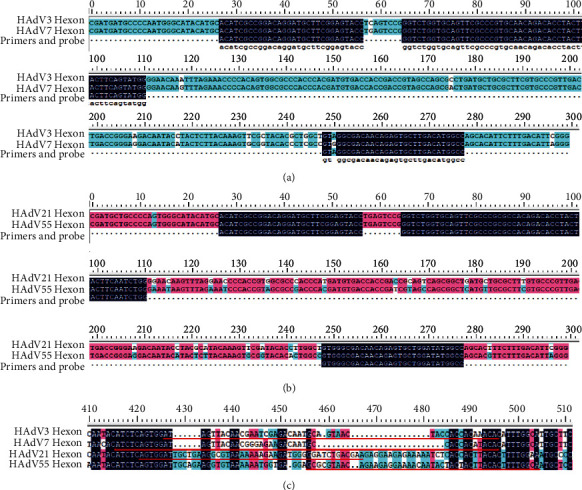
Primers and probes designed for RPA assay. (a) Primer and probe sequences (dark blue) screened from hexon genes of HAdV 3 and HAdV 7 for RPA-HAdV; (b) primer and probe sequences (dark blue) screened from hexon genes of HAdV 21 and HAdV 55 for RPA-HAdV; (c) probes (red underlined) screened for typing RPA assay.

Primers and probes were designed in the target sequences. In RPA-HAdV, the nucleic acid sequence from base 1 to 300 of hexon gene in each serotype, which shared an identity rate of 95.6%, was selected as the target sequence (Figures 2(a) and 2(b)). Actually, it is hard to screen a probe around 46 bp that is exactly identical among the four serotypes. So 2 probes with 4 bases of diversity separately from HAdV 3/7 and HAdV 21/55 were designed for further screening (Table 1). One common forward primer and 2 unique reverse primers were designed (Table 1). The 2 reverse primers, which were from HAdV 3/7 and HAdV 21/55, respectively, had 3 bases of diversity.

In the typing RPA assays, specific probes were screened firstly using DNAman as shown on Figure 2(c) (sequences underlined). The probes varied a lot in sequences. Three forward primers and 3 reverse primers upstream and downstream the probe were designed as shown in Table 1.

### 3.3. Screening of the Best Primer and Probe Combinations

**Figure 3:**
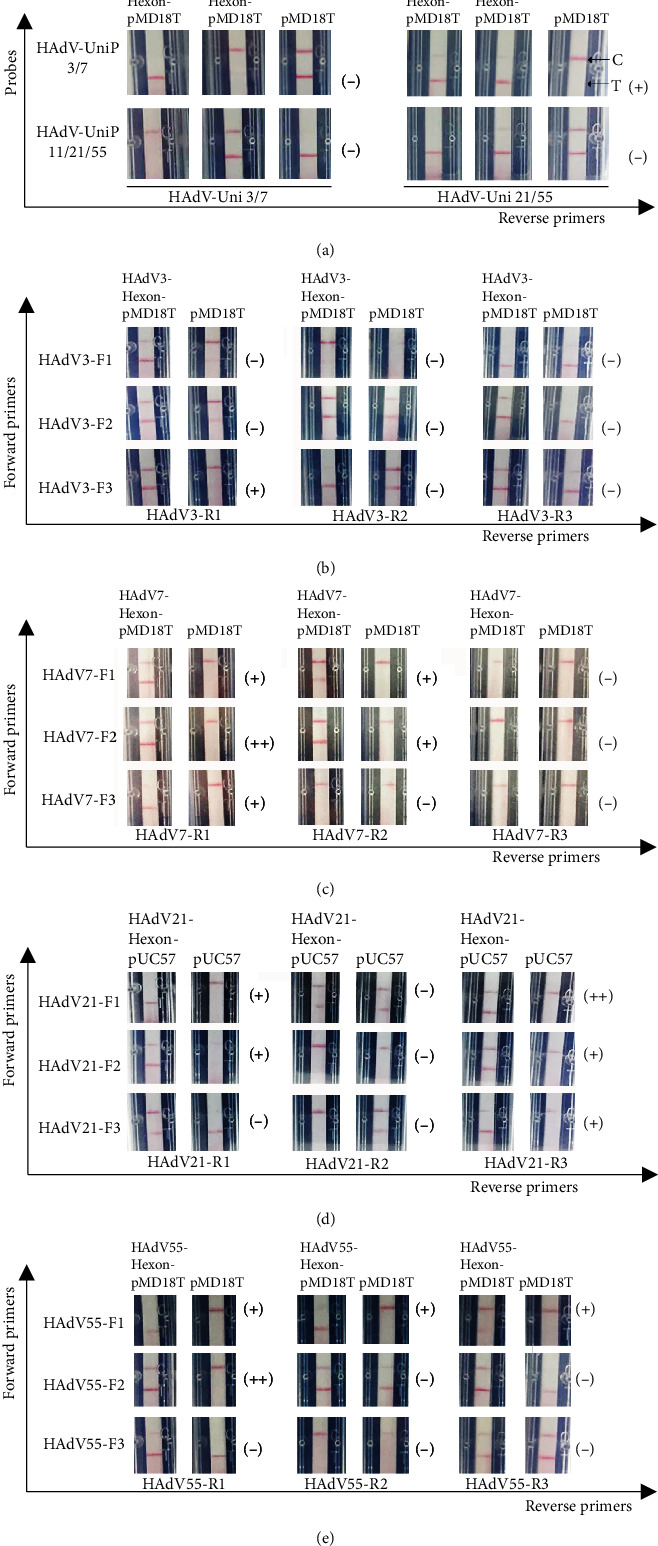
Evaluation of various combinations of forward primer, reverse primer, and probe in (a) RPA-HAdV, (b) RPA-HAdV3, (c) RPA-HAdV7, (d) RPA-HAdV21, and (e) RPA-HAdV55. The primers, probes, and templates used here are indicated. The top and bottom red lines on each strip are the control (C) and test (T) lines, respectively. (−): bad result; (+): good result; (++): excellent result.

Several sets of primers or probes were designed for each RPA assay as shown in Table 1, and the best combination was screened. As shown on Figure 3(a), the second group, using HAdV-UniP3/7 as probe, HAdV-UniF as forward primer, and HAdV-UniR21/55 as reverse primer, could detect both positive plasmids without detecting the control plasmid. This group of primers and probe was screened to establish the RPA-HAdV assay for all the four serotypes.

For typing methods, the best combinations were screened as HAdV3-F3&HAdV3-R1 for HAdV 3 (Figure 3(b)), HAdV7-F2&HAdV7-R1 for HAdV 7 (Figure 3(c)), HAdV21-F1&HAdV21-R3 for HAdV 21 (Figure 3(d)), and HAdV55-F2&HAdV55-R1 for HAdV 55 (Figure 3(e)). The RPA-LF assays using these primers and probes could detect the corresponding positive plasmid with deep dark color on the T line of the strips, without recognizing the control plasmid.

### 3.4. Optimization of Reaction Conditions

**Figure 4:**
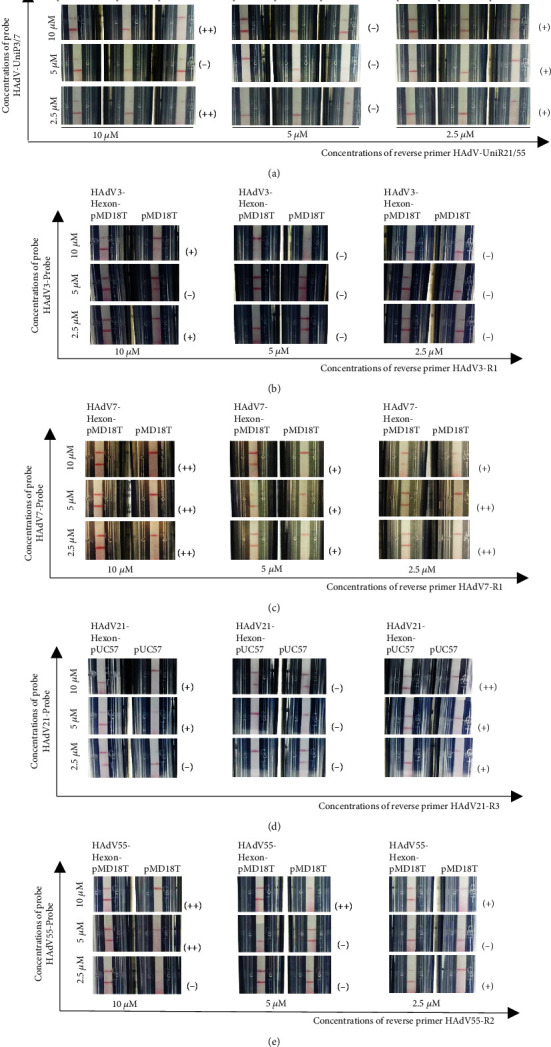
Optimization of reverse primer and probe concentrations in reaction systems of (a) RPA-HAdV, (b) RPA-HAdV3, (c) RPA-HAdV7, (d) RPA-HAdV21, and (e) RPA-HAdV55. The concentrations of reverse primers and probes used here are indicated. The top and bottom red lines on each strip are the control and test lines, respectively. (−): bad result; (+): good result; (++): excellent result.

The concentrations of reverse primers or probes in the established RPA-LF methods were optimized (Figure 4). When groups with various concentrations of reverse primers or probes in each assay performed modest results, the group with lower concentration of probe was favored considering the cost. In RPA-HAdV, 2.5 *μ*M of HAdV-UniP3/7 and 10 *μ*M of HAdV-UniR21/55 were favored to establish the ultimate reaction system (Figure 4(a)). In the typing RPA-LF methods, 2.5 *μ*M of HAdV3-Probe and 10 *μ*M of HAdV3-R1 in RPA-HAdV3 (Figure 4(b)), 2.5 *μ*M of HAdV7-Probe and 2.5 *μ*M of HAdV7-R1 in RPA-HAdV7 (Figure 4(c)), 2.5 *μ*M of HAdV21-Probe and 2.5 *μ*M of HAdV21-R3 in RPA-HAdV21 (Figure 4(d)), and 5 *μ*M of HAdV55-Probe and 10 *μ*M of HAdV55-R1 in RPA-HAdV55 (Figure 4(e)) were favored.

**Figure 5:**
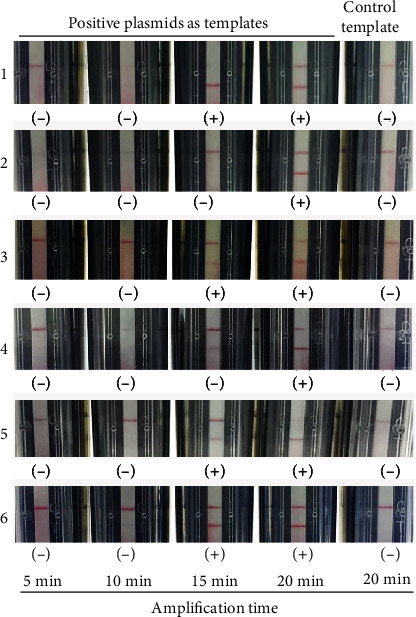
Optimization of amplification time of RPA-HAdV in detecting plasmid HAdV3-Hexon-pMD18T (1) and HAdV55-Hexon-pMD18T (2) and of RPA-HAdV3 (3), RPA-HAdV7 (4), RPA-HAdV21 (5), and RPA-HAdV55 (6) in detecting their corresponding positive plasmids. The top and bottom red lines on each strip are the control and test lines, respectively. Various amplification times are indicated. (−): negative result; (+): positive result.

Various amplification times for each assay were tested, and the shortest one with modest result was determined as the best amplification time. As shown on Figure 5, the best amplification time was 15 and 20 min for RPA-HAdV to detect plasmid HAdV3-Hexon-pMD18T and HAdV55-Hexon-pMD18T, respectively. So the best amplification time of RPA-HAdV was confirmed to be 20 min. The best amplification time of the typing RPA-LF methods was 15 min, except RPA-HAdV7, the best time of which was 20 min.

### 3.5. Detection Limit Evaluation

**Figure 6:**
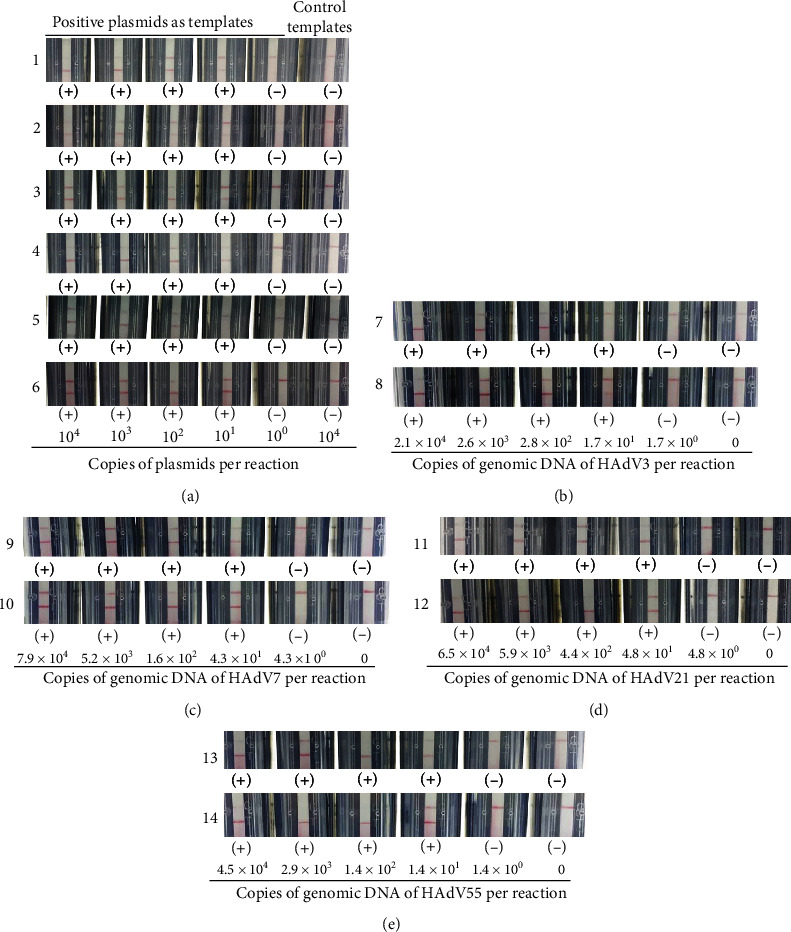
Evaluation of the detection limit of each RPA-LF assay in detecting (a) positive plasmids or genomic DNA of (b) HAdV 3, (c) HAdV 7, (d) HAdV 21, and (e) HAdV 55. 1, 2, 7, 9, 11, and 13 represent detection results of various concentrations of plasmids HAdV3-Hexon-pMD18T and HAdV55-Hexon-pMD18T as well as genomic DNA of HAdV 3, HAdV 7, HAdV 21, and HAdV 55 with the HAdV-RPA method, respectively; 3, 4, 5, and 6 represent detection results of various concentrations of positive plasmids with HAdV3-RPA, HAdV7-RPA, HAdV21-RPA, and HAdV55-RPA methods, respectively; 8, 10, 12, and 14 represent detection results of various concentrations of genomic DNA with HAdV3-RPA, HAdV7-RPA, HAdV21-RPA, and HAdV55-RPA methods, respectively. The top and bottom red lines on each strip are the control and test lines, respectively. The concentrations of the templates are indicated. (−): negative result; (+): positive result.

The detection limit for each RPA-LF assay was evaluated. As a result, RPA-HAdV could detect 10 copies of plasmid HAdV3-Hexon-pMD18T or HAdV55-Hexon-pMD18T (Figure 6(a)), 280 copies of genomic DNA of HAdV 3 (Figure 6(b)), 43 copies of genomic DNA of HAdV 7 (Figure 6(c)), 48 copies of genomic DNA of HAdV 21 (Figure 6(d)), and 14 copies of genomic DNA of HAdV 55 (Figure 6(e)) per reaction. The detection limits of RPA-HAdV3, RPA-HAdV7, RPA-HAdV21, and RPA-HAdV55 were 10, 10, 100, and 10 copies per reaction in detection of corresponding positive plasmids, respectively (Figure 6(a)), and were 17, 43, 48, and 14 copies per reaction (Figures 6(b)–6(e)) in detecting corresponding genomic DNA, respectively.

### 3.6. Sensitivity and Specificity Analysis

**Figure 7:**
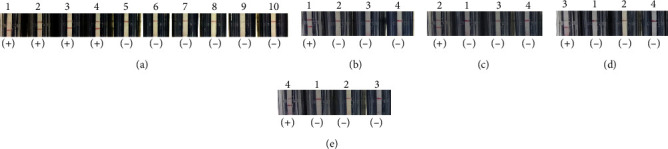
Specificity analysis of (a) RPA-HAdV, (b) RPA-HAdV3, (c) RPA-HAdV7, (d) RPA-HAdV21, and (e) RPA-HAdV55 in detecting DNA of various pathogens. Genomic DNA of HAdV 3 (1), HAdV 7 (2), HAdV 21 (3), HAdV 55 (4), *Chlamydia psittaci* (5), *Coxiella burnetii* (6), *Streptococcus suis* (7), *Escherichia coli* (8), and *Staphylococcus aureus* (9) as well as control plasmid pUC57 (10) were used as templates. The top and bottom red lines on each strip are the control and test lines, respectively. (−): negative result; (+): positive result.

Genomic DNA or RNA purified from 200 clinical samples of HAdV 3, HAdV 7, HAdV 55, and influenza virus A (H1 or H3 positive)-infected patient, as well as 10 HAdV 21 DNA-spiked samples, which were determined using real-time PCR/RT-PCR, were used to measure the sensitivity and specificity of the RPA-LF methods. As shown in Table 2, the sensitivities of RPA-HAdV3, RPA-HAdV7, RPA-HAdV21, RPA-HAdV55, and RPA-HAdV were 100%, 92%, 100%, 100%, and 93%, respectively. The specificities of RPA-HAdV3, RPA-HAdV7, RPA-HAdV21, RPA-HAdV55, and RPA-HAdV were 98%, 100%, 99%, 99%, and 100%, respectively. RPA-HAdV method could detect these four serotypes without recognizing *C. psittaci*, *C. burnetii*, *S. suis*, *E. coli*, and *S. aureus*, while the other methods could detect their corresponding genomic DNA without detecting those of the other serotypes of HAdV (Figure 7).

## 4. Discussion

HAdV epidemics often break out in clusters in schools or army. Accurate and prompt detection and typing of HAdV are highly in demand to guide antiviral treatment and reduce the disease severity [3, 16]. In this study, simple, fast, visual, sensitive, and specific detection methods based on RPA-LF assay towards four serotypes of HAdV species B were established and evaluated.

Hexon gene is one of the highly conserved genes in HAdV. Actually, most of the nucleic acid detection methods towards HAdV were designed based on the hexon or fiber gene in previous studies [26–30]. In the RPA assay, the lengths of primer and probe required were around 30 bp and 46 bp, respectively, which were much longer than those used in the real-time PCR assay. An identical nucleic acid sequence of 46 bp among the four serotypes of HAdV was hard to find, while it is easier between two serotypes. So, in the establishment of RPA-HAdV, the similar reverse primer or probe sequence, which performed 3 to 4 bases diversity between HAdV 3/7 and HAdV 21/55, was synthesized separately and evaluated their performance in detection of various positive plasmids. Considering that HAdV 3 and 7 or HAdV 21 and 55 share the same primer and probe sequences, positive plasmids only HAdV3-Hexon-pMD18T and HAdV55-Hexon-pMD18T but not HAdV7-Hexon-pMD18T or HAdV21-Hexon-pUC57 were used in the evaluation. A probe from HAdV 3/7 (HAdV-UniP3/7) and a reverse primer from HAdV 21/55 (HAdV-UniR21/55) were determined as the best combination to detect all the four serotypes. This indicates the RPA assay is tolerant to the variation of several bases in primer and probe sequence. In addition, using degenerate primer and probe is also an option.

In RPA-LF method, the FAM- and biotin-labeled amplified product, which was generated by biotin-labeled reverse primer and FAM-labeled probe, would develop the T line of the lateral flow strip in the cassette. Then, the result could be judged by the naked eyes. The concentrations of reverse primer and probe but not forward primer were optimized, considering their more important roles in development of strips as well as higher cost. Also, the amplification time was optimized for each assay, and 15 or 20 min was acceptable.

The detection limit of all the established methods except RPA-HAdV21 was 10 copies of positive plasmids per reaction, which was comparable to qPCR. RPA-HAdV could detect 280, 43, 48, and 14 copies of genomic DNA of HAdV 3, HAdV 7, HAdV 21, and HAdV 55 per reaction, respectively. The detection limits of RPA-HAdV3, RPA-HAdV7, RPA-HAdV21, and RPA-HAdV55 were 17, 43, 48, and 14 copies per reaction, respectively, in detecting corresponding genomic DNA. Compared with the qPCR method used in the present study, the RPA-HAdV performed similar detection limit in detecting genomic DNA of HAdV 7 or HAdV 21 (43 and 48 copies/reaction in qPCR, respectively), higher detection limit in detecting genomic DNA of HAdV 3 (17 copies/reaction in qPCR), and lower detection limit in detecting genomic DNA of HAdV 55 (140 copies/reaction in qPCR). However, the RPA-HAdV3, RPA-HAdV7, or RPA-HAdV21 performed similar detection limit, and the RPA-HAdV55 performed lower detection limit in detecting corresponding genomic DNA. Considering the viral loads of various serotypes of HAdV in throat swabs ranged from 5 × 10^5^ to 1.5 × 10^9^ copies/mL (about 2.5 × 10^3^ to 7.5 × 10^7^ copies/*μ*L of genomic DNA after extraction using a MiniBEST Viral RNA/DNA Extraction Kit) reported in previous publication [20], the established methods could detect the responding HAdV effectively.

The established methods performed modest sensitivities (ranging from 92% to 100%) and specificities (ranging from 98% to 100%) evaluated using limited clinical samples. Several HAdV 7-positive samples being not detected may be due to the low viral load after long-term storage, which influenced the sensitivities. The established methods did not recognize genomic DNA from other pathogens used in the study. Only 1 or 2 HAdV 7-positive samples were recognized by other serotyping methods, which may be caused by contamination in some steps. However, more clinical samples of various HAdV serotypes should be used to evaluate the methods, and other adenoviral species or serotypes should be used to evaluate the serotype-specific RPA-LF assays. Also, in practical application, an internal control should be introduced to eliminate potential inhibitor of the method in samples, and a DNA extraction method suitable to field sites or resource-limited sites should be developed. In addition, a multiple serotyping method based on RPA-LF may be more useful.

Our assays can be used to determine whether or not a sample is positive or negative for ARD-causing species B of HAdV and identify HAdV 3, 7, 21, and 55 in clinical samples. Compared with the traditional PCR or real-time PCR methods, which usually consume 2 h and need expensive machines [20], the established RPA-LF methods here could complete in less than 25 min with RPA assay consuming 15 to 20 min and LF test consuming 5 min without any expensive instrument, while performing similar or higher sensitivity. Our methods are more suitable for use in the field and resource-limited areas. When future HAdV outbreaks occur in crowds such as army or schools, we will have the tools available in the field to rapidly determine which adenovirus is causing the infection and treat the outbreaks faster and more targeted.

In conclusion, we successfully established universal and typing methods for detection of HAdV 3, HAdV 7, HAdV21, and HAdV 55 based on RPA assay and LF test, which were simple, sensitive, and specific. The method had a detection limit of 10 to 280 copies/reaction in detecting corresponding positive plasmid or genomic DNA. The sensitivity and specificity were >92% and >98%, respectively. The RPA-LF methods are promising for a wide use in the field and resource-limited area, considering they can complete detection in 25 min with a cheap constant temperature equipment, though more clinical patient samples are needed to evaluate the methods in the future.

## Figures and Tables

**Table 1:** The designed primers and probes for RPA and PCR.

Usage	Type	ID	Sequence (5′-3′)
RPA-HAdV	Forward primer	HAdV-UniF	ACATCGCCGGACAGGATGCTTCGGAGTACC
	Reverse primer	HAdV-UniR3/7	Biotin-GGCCATGTCAAGCACTCTGTTGTCGCCTAC
	Reverse primer	HAdV-UniR21/55	Biotin-GGCCATATCCAGCACTCTGTTGTCGCCCAC
	Probe	HAdV-UniP3/7	FAM-GGTCTGGTGCAGTTCGCCCGTGCAACAGAC-[THF]-CCTACTTCAGTATGG-PO4
	Probe	HAdV-UniP21/55	FAM-GGTCTGGTGCAGTTCGCCCGCGCCACAGAC-[THF]-CCTACTTCAATCTGG-PO4
RPA-HAdV3	Forward primer	HAdV3-F1	ACCGGGAAGACAATACCTACTCTTACAAAG
	Forward primer	HAdV3-F2	ACGCTGGCTGTAGGCGACAACAGAGTGCTT
	Forward primer	HAdV3-F3	GCCATATTCCGGCACAGCTTACAATTCACT
	Reverse primer	HAdV3-R1	Biotin-TTCTTCTCCAACTTGAGGCTCTGGCTGATA
	Reverse primer	HAdV3-R2	Biotin-TTGTCTCCCTTCATGGAAGCAATGCCAAAT
	Reverse primer	HAdV3-R3	Biotin-GTAGCATGGCTTCATGTTGGTAGCTGGTTT
	Probe	HAdV3-Probe	FAM-ATAGTTACAACGAATCGAGACAATGCAGTA-[THF]-CTACCACCACAAACA-PO_4_
RPA-HAdV7	Forward primer	HAdV7-F1	TGACCACCGACCGTAGCCAGCGACTGATGC
	Forward primer	HAdV7-F2	GCCATATTCCGGCACAGCTTACAATTCACT
	Forward primer	HAdV7-F3	ATACATACTCTTACAAGTGCGGTACACCC
	Reverse primer	HAdV7-R1	Biotin-TTCTTCTCCAACTTGAGGCTCTGGCTGATA
	Reverse primer	HAdV7-R2	Biotin-TTCAACATCTCCTTCGGTTGGTGTTACTTT
	Reverse primer	HAdV7-R3	Biotin-CTACAAAGTTATCCCTGAAGCCAATGTAAT
	Probe	HAdV7-Probe	FAM-TCTCAGTGGATAGTTACAACGGGAGAAGAC-[THF]-ATGCCACCACATACA-PO_4_
RPA-HAdV21	Forward primer	HAdV21-F1	TTTGTGCCCGTTGACCGGGAAGACAATACC
	Forward primer	HAdV21-F2	AATACCTACGCATACAAAGTTCGATACACC
	Forward primer	HAdV21-F3	ATACAAAGTTCGATACACCTTGGCTGTGGG
	Reverse primer	HAdV21-R1	Biotin-TGCATAAATTGGTTTGGCTTCGCCGTCTGT
	Reverse primer	HAdV21-R2	Biotin-TCCCACCTGAGGTTCTGGTTGGTATAGTTT
	Reverse primer	HAdV21-R3	Biotin-GCTCTACCACCATACTTCTCAGTTGTTCCA
	Probe	HAdV21-Probe	FAM-AGTGGATTGCTGAAGGCGTAAAAAAAGAAG-[THF]-TGGGGGATCTGACGA-PO4
RPA-HAdV55	Forward primer	HAdV55-F1	GCTCCTAAAGGCGCTCCAAATACATCTCAG
	Forward primer	HAdV55-F2	TCAAACCCTATTCTGGTACGGCTTACAACT
	Forward primer	HAdV55-F3	CCGTTGACCGGGAGGACAATACATACTCTT
	Reverse primer	HAdV55-R1	Biotin-CGACTTTCTGATTTGGCTGCTCCGTTGTTT
	Reverse primer	HAdV55-R2	Biotin-CCATCAAGGTCAGTCCAAGTTTCATCTCCC
	Reverse primer	HAdV55-R3	Biotin-AACTTTCAAACCTATTGGGAGTCCTTCTTT
	Probe	HAdV55-Probe	FAM-CGCGTAACAGAAGAGGAAAACAATACTACT-[THF]-CTTACACTTTTGGCA-PO_4_
PCR for HAdV 3 (2835 bp)	Forward primer	Hexon3-F	CGAGGCTGAGTTGCTTTCA
	Reverse primer	Hexon3-R	TCGGACGATGGCTTTGAG
PCR for HAdV 7 (2814 bp)	Forward primer	Hexon7-F	CGAGGCTGAGTTGCTTTCA
	Reverse primer	Hexon7-R	TCGGACGATGGCTTTGAG
PCR for HAdV 55 (2847 bp)	Forward primer	Hexon55-F	CGACGCTGAGTTACTTTCA
	Reverse primer	Hexon55-R	TTGGACAATGGCTCTGAG
Quantitative PCR	Forward primer	qPCR-F	ATGGCCACCCCATCGAT
	Reverse primer	qPCR-R	ACTCAGGTACTCCGAAGCATCCT
	Probe	qPCR-P	FAM-TGGGCATACATGCACATCGCCG-BHQ1

**Table 2:** Sensitivity and specificity analysis of the established methods.

Samples	Methods	RPA-HAdV3	RPA-HAdV7	RPA-HAdV21	RPA-HAdV55	RPA-HAdV
HAdV 3 positive (*n* = 15)	Positive	15	0	0	0	14
	Negative	0	15	15	15	1
	Sensitivity	100%	—	—	—	93%
	Specificity	—	100%	100%	100%	—
HAdV 7 positive (*n* = 89)	Positive	2	82	1	1	81
	Negative	87	7	88	88	8
	Sensitivity	—	92%	—	—	91%
	Specificity	98%	—	99%	99%	—
HAdV 21 positive (*n* = 10)	Positive	0	0	10	0	10
	Negative	10	10	0	10	0
	Sensitivity	—	—	100%	—	100%
	Specificity	100%	100%	—	100%	—
HAdV 55 positive (*n* = 17)	Positive	0	0	0	17	17
	Negative	17	17	17	0	0
	Sensitivity	—	—	—	100%	100%
	Specificity	100%	100%	100%	—	—
Influenza A (H1) positive (*n* = 41)	Positive	—	—	—	—	0
	Negative	—	—	—	—	41
	Sensitivity	—	—	—	—	—
	Specificity	—	—	—	—	100%
Influenza A (H3) positive (*n* = 38)	Positive	—	—	—	—	38
	Negative	—	—	—	—	0
	Sensitivity	—	—	—	—	—
	Specificity	—	—	—	—	100%
Overall	Sensitivity (95% confidence interval)	100% (0.75-1, *n* = 131)	92% (0.84-0.97, *n* = 131)	100% (0.66-1, *n* = 131)	100% (0.77-1, *n* = 131)	93% (87%-97%, *n* = 210)
	Specificity (95% confidence interval)	98% (0.93-1) (*n* = 131)	100% (0.90-1) (*n* = 131)	99% (0.95-1) (*n* = 131)	99% (0.94-1) (*n* = 131)	100% (0.94-1) (*n* = 210)

## Data Availability

The data used to support the findings of this study are included within the article.
